# Neural dysfunction during temporal discounting in paediatric Attention-Deficit/Hyperactivity Disorder and Obsessive-Compulsive Disorder

**DOI:** 10.1016/j.pscychresns.2017.09.008

**Published:** 2017-11-30

**Authors:** Luke J. Norman, Christina O. Carlisi, Anastasia Christakou, Kaylita Chantiluke, Clodagh Murphy, Andrew Simmons, Vincent Giampietro, Michael Brammer, David Mataix-Cols, Katya Rubia

**Affiliations:** a Department of Child and Adolescent Psychiatry, Institute of Psychiatry, Psychology and Neuroscience, King's College London, UK; b Department of Psychiatry, University of Michigan, Ann Arbor, Michigan, USA; c Centre for Integrative Neuroscience and Neurodynamics, School of Psychology and Clinical Language Sciences, University of Reading, UK; d Sackler Institute for Translational Neurodevelopment and Department of Forensic and Neurodevelopmental Sciences, Institute of Psychiatry, Psychology and Neuroscience, King׳s College London, London, UK; e Department of Neuroimaging, Institute of Psychiatry, Psychology and Neuroscience, King's College London, UK; f National Institute for Health Research (NIHR) Biomedical Research Centre (BRC) for Mental Health at South London and Maudsley NHS Foundation Trust and Institute of Psychiatry, Psychology & Neuroscience, King's College London, UK; g Department of Neurobiology, Care Sciences and Society, Center for Alzheimer Research, Division of Clinical Geriatrics, Karolinska Institutet, Stockholm, Sweden; h Department of Clinical Neuroscience, Karolinska Institutet, Stockholm, Sweden

**Keywords:** ADHD, OCD, fMRI, Discounting, Reward, Impulsivity

## Abstract

Both Attention-Deficit/Hyperactivity Disorder (ADHD) and Obsessive-Compulsive Disorder (OCD) are associated with choice impulsivity, i.e. the tendency to prefer smaller immediate rewards over larger delayed rewards. However, the extent to which this impulsivity is mediated by shared or distinct underlying neural mechanisms is unclear. Twenty-six boys with ADHD, 20 boys with OCD and 20 matched controls (aged 12–18) completed an fMRI version of an individually adjusted temporal discounting (TD) task which requires choosing between a variable amount of money now or £100 in one week, one month or one year. Activations to immediate and delayed reward choices were compared between groups using a three-way ANCOVA. ADHD patients had steeper discounting rates on the task relative to controls. OCD patients did not differ from controls or patients with ADHD. Patients with ADHD and OCD showed predominantly shared activation deficits during TD in fronto-striato-insular-cerebellar regions responsible for self-control and temporal foresight, suggesting that choice impulsivity is mediated by overlapping neural dysfunctions in both disorders. OCD patients alone showed dysfunction relative to controls in right orbitofrontal and rostrolateral prefrontal cortex, extending previous findings of abnormalities in these regions in OCD to the domain of choice impulsiveness.

## Highlights

• ADHD patients alone showed significantly steeper discounting relative to controls.
• ADHD and OCD shared underactivation in fronto-striato-insular-cerebellar regions during delayed choices.
• Only patients with OCD had dysfunction in right orbitofrontal and rostrolateral prefrontal cortex relative to controls.

## 1 Introduction

Impulsivity is a multifaceted construct and is typified by a premature, poorly controlled, delay averse response pattern where the consequences of acts are poorly considered (Fineberg et al., 2014, Rubia, 2002, Rubia et al., 2009). It has been investigated as a potential correlate of both Attention-Deficit/Hyperactivity Disorder (ADHD) and Obsessive-Compulsive Disorder (OCD), disorders which affect around 3–8% and 1–3% of children respectively, as well as around 4% (ADHD) and 2% (OCD) of adults (Biederman et al., 2012, Ruscio et al., 2010). A role for impulsivity has been hypothesised in both disorders. This is despite their very distinct symptom profiles, with ADHD defined by age-inappropriate problems with inattention, impulsivity and hyperactivity, and OCD defined by obsessions, i.e. recurrent and intrusive thoughts (e.g., on themes of contamination, checking, orderliness and symmetry), and compulsions, i.e. repetitive, ego-dystonic and time-consuming behavioural and mental rituals (e.g., repetitive washing or checking) (American Psychiatric Association, 2013).

Impulsivity is a core feature of ADHD, which is particularly prevalent during childhood and adolescence (American Psychiatric Association, 2013). In OCD, increased self-reported impulsivity is associated with poorer treatment outcomes (Kashyap et al., 2012). Compulsivity and impulsivity are traditionally situated at opposing ends of a compulsivity-impulsivity spectrum, with OCD considered the archetypal disorder of compulsivity (Fineberg et al., 2014, Robbins et al., 2012). However, recent research questions this model and suggests that compulsivity and impulsivity may in fact co-exist in OCD (Fineberg et al., 2014, Grassi et al., 2015, Kashyap et al., 2012, Sohn et al., 2014). In particular, impulsivity may underlie a tendency to perform OCD behaviours in order to bring about an initially rewarding outcome (i.e., relieve anxiety) despite their negative long-term consequences (Fontenelle et al., 2015, Grassi et al., 2015).

To date, most neuroimaging studies of impulsivity in ADHD and OCD have focused on tasks involving motor impulsivity, i.e. the poor ability to inhibit inappropriate prepotent responses during response inhibition paradigms (Benzina et al., 2016, Lipszyc and Schachar, 2010, Norman et al., 2016, Rubia et al., 2010, Woolley et al., 2008, Zandt et al., 2009). Recent fMRI studies and a large meta-analysis have provided evidence for disorder-specific fronto-insula-striatal activation abnormalities in the two disorders, with lateral inferior prefrontal underactivation being disorder-specific to ADHD (Norman et al., 2016, Norman et al., 2017, Rubia et al., 2010, Rubia et al., 2011), and medial frontal dysfunction being disorder-specific to OCD (Norman et al., 2016, Norman et al., 2017). However, impulsivity is a multifaceted construct (Fineberg et al., 2014), and much less research has examined the neural basis of other impulsivity domains such as choice impulsivity, defined as the tendency to prefer smaller immediate rewards over larger delayed rewards, which is also a feature of ADHD and OCD (Noreika et al., 2013, Patros et al., 2016, Sohn et al., 2014).

Choice impulsivity is typically measured in temporal discounting (TD) tasks (Christakou et al., 2011, Fineberg et al., 2014, Hamilton et al., 2015), during which participants are provided with a series of choices between small immediate rewards and larger rewards available after a hypothetical delay, typically ranging from weeks to years. TD refers to the finding that the subjective values of rewards available after a delay decrease as a function of the length of the delay (Christakou et al., 2011, Hamilton et al., 2015). In studies incorporating adjusting-amount procedures, adjustments of the immediate reward are performed according to the individual participant's previous choices using an online algorithm, such that the range of options is narrowed around the point where the subjective value of the immediate reward is equal to that of the fixed delayed reward (the indifference point) (Carlisi et al., 2016, Christakou et al., 2011, Richards et al., 1997). Indifference points across different delay lengths are used to produce a discounting curve, which is typically hyperbolic (i.e., as delay periods become longer, the rate at which reward values are declined decreases more drastically) (Peters and Buchel, 2011). The steepness of discounting curves varies widely between individuals, and steeper discounting indicates more impulsive choices (Hamilton et al., 2015, Peters and Buchel, 2011). The task measures several cognitive functions, such as the inhibition of the immediate thrill of the reward, the sensitivity of an individual to the varying real or hypothetical delay of time in units of reward (delay aversion), temporal foresight to understand the future gain of the delayed choice, as well as inter-temporal decision making and reward evaluation with respect to its delay (Christakou et al., 2011, Hamilton et al., 2015, Noreika et al., 2013, Rubia et al., 2009).

Performance during TD tasks relies on two main brain networks (Christakou et al., 2011, Hare et al., 2014, Peters and Buchel, 2011). The first of these involves the ventral striatum, ventromedial prefrontal cortex/orbitofrontal cortex (VMPFC, OFC), and posterior cingulate cortex (PCC), i.e. paralimbic regions involved in processing rewards and motivation (Christakou et al., 2011, Peters and Buchel, 2011). The second network involves inferior, rostrolateral and dorsolateral prefrontal cortex (IFG, RLPFC, DLPFC), anterior insula (AI), dorsal striatum, parietal lobe and cerebellum, i.e. regions involved in executive functions such as inhibitory control (Hart et al., 2013, Wesley and Bickel, 2014), working memory (Nee et al., 2013, Wesley and Bickel, 2014), planning (van den Heuvel et al., 2003), prospection (Burgess et al., 2011), reappraisal (Delgado et al., 2008, Giuliani et al., 2014, Kober et al., 2010, Volkow et al., 2010), time estimation (Hart et al., 2012, Noreika et al., 2013, Rubia et al., 2009) and attentional control (Hart et al., 2013, Rubia et al., 2011), processes which are important for making farsighted delayed choices (Christakou et al., 2011, Rubia et al., 2009, Wesley and Bickel, 2014). ADHD patients have shown steeper discounting rates than controls in TD tasks and reduced activation in executive function regions including IFG, AI and dorsal striatum during delayed choices (Carlisi et al., 2016, Rubia et al., 2009), as well as altered correlations between IFG, temporal lobe, AI, supplementary motor area and cerebellum activation and TD discounting rates relative to controls (Chantiluke et al., 2014). In adult ADHD, reduced activation has been reported in DLPFC, striatal, parietal and cerebellar regions during TD (Ortiz et al., 2015, Plichta et al., 2009).

Impulsive decision making is also a feature of OCD (Cavedini et al., 2002, Kashyap et al., 2012, Sohn et al., 2014), including during TD (Sohn et al., 2014). Previous research has established the importance of VMPFC/OFC and striatal regions in OCD (Menzies et al., 2008, Radua and Mataix-Cols, 2009, Radua et al., 2010, Saxena and Rauch, 2000), which are reliably activated during symptom provocation (Rotge et al., 2008), dysfunctional during cognitive and reward tasks (Page et al., 2009, Remijnse et al., 2009), and highly relevant to TD (Christakou et al., 2011, Peters and Buchel, 2011). In line with this, we recently showed that adolescents with OCD had altered activation in VMPFC/OFC and left caudate during TD, as well as in DLPFC, IFG, AI, parietal lobes and cerebellum (Carlisi et al., 2017b).

It is not clear to what extent the underlying brain mechanisms of TD differ or are shared between disorders as no published studies have directly compared ADHD and OCD patients during TD using fMRI. Shared neural dysfunction would lend credence to the idea that impulsive decision making in ADHD and OCD is a shared transdiagnostic mechanism, whereas disorder-specific patterns of functional abnormalities would suggest that shared decision making impairments are similar phenocopies associated with distinct underlying mechanisms (Robbins et al., 2012).

The aim of this study was therefore to conduct the first direct comparison of the neurofunctional substrates of TD in ADHD and OCD patients using fMRI. Behaviourally, we anticipated that both patient groups would show steeper discounting relative to controls (Patros et al., 2016, Sohn et al., 2014). In the brain, we hypothesised shared underactivation in both disorders relative to controls in striatal, DLPFC and cerebellar regions previously implicated in ADHD (Carlisi et al., 2016, Hart et al., 2013, Norman et al., 2016) and OCD (Carlisi et al., 2017a, Carlisi et al., 2017b, Carlisi et al., in press; Norman et al., 2016; Rubia et al., 2010, Rubia et al., 2011). We, however, hypothesised more prominent or disorder-specific abnormalities in OCD patients in VMPFC/OFC (Carlisi et al., 2017b, Menzies et al., 2008, Norman et al., 2016, Norman et al., 2017), and larger or disorder-specific underactivation in IFG in ADHD patients (Noreika et al., 2013, Norman et al., 2016, Norman et al., 2017, Rubia et al., 2009, Rubia et al., 2010). Data have been published in other forms elsewhere, although in this paper we provide the novel comparison between controls, patients with ADHD and patients with OCD (Carlisi et al., 2016, Carlisi et al., 2017b, Chantiluke et al., 2014, Christakou et al., 2011, Rubia et al., 2009).

## 2 Methods

### 2.1 Participants

Sixty-six (26 ADHD, 20 OCD, 20 controls) right handed (Oldfield, 1971) male adolescents participated, aged between 12–18, and with an IQ>70 as measured by the Wechsler Abbreviated Scale of Intelligence-Revised (WASI-R) short form (Wechsler, 2008). ADHD boys were recruited from local child and adolescent mental health services (CAMHS) and met DSM-IV criteria for inattentive/hyperactive-impulsive combined subtype, as assessed using the standardized Maudsley diagnostic interview (Goldberg and Murray, 2006), and scored above clinical cut-off on the Conner's Parent Rating Scale-Revised (CPRS-R) (Conners et al., 1998) and the inattention/hyperactivity scale of the parent Strength and Difficulty Questionnaire (SDQ) (Goodman, 1997). A consultant psychiatrist excluded comorbidity with other disorders (except conduct disorder), including OCD. Twelve ADHD boys were medication naïve. Fourteen ADHD boys were receiving psychostimulant medication and underwent a 48 h washout period prior to scanning. OCD boys were recruited from a national specialist clinic for child and adolescent OCD and local CAMHS and had clinical diagnoses of OCD, assessed according to the 10th edition of the International Classification of Diseases (ICD-10) criteria and the Children's Yale-Brown Obsessive Compulsive Scale (Scahill et al., 1997). Absence of comorbidity, including comorbid ADHD, was confirmed by a consultant psychiatrist. Sixteen OCD boys were medication naïve. Four were being treated with selective serotonin re-uptake inhibitor (SSRI) medication. One of these four patients was receiving risperidone as an augmentation treatment. Control participants had no psychiatric diagnoses, and were recruited using local advertising. Only boys were studied due to the preponderance of males in adolescent ADHD and OCD populations, and to achieve greater homogeneity across participants and groups (Geller et al., 1998, Willcutt, 2012). Exclusion criteria included comorbid psychiatric disorders, medical disorders affecting brain development, drug/alcohol dependency, head injury, abnormal brain structural MRI findings and MRI contraindications.

Ethical approval was obtained from the local Research Ethics Committee (05/Q0706/275), and the study was conducted in accordance with the Declaration of Helsinki. Study details were explained to both child and guardian. Written informed consent was obtained for all participants.

### 2.2 Temporal discounting fMRI task

**Fig. 1:**
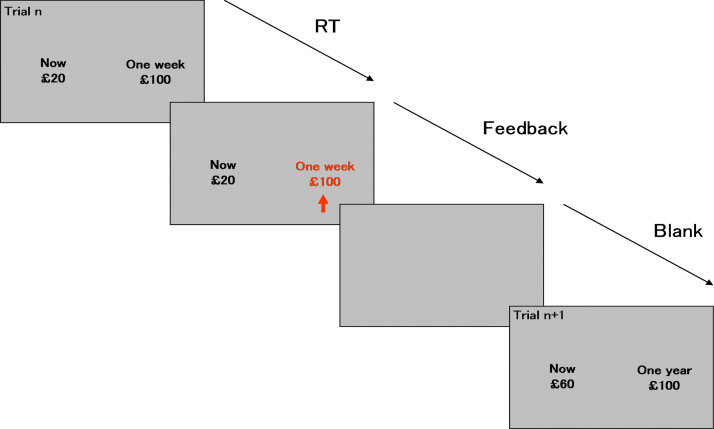
*Schematic representation of the temporal discounting (TD) task.* In the TD task, participants choose between an amount of money (£100) available after a delay of one week, one month and one year or a smaller amount of money available immediately (0–£100). For each participant, an algorithm is used to find values for the immediate option which are subjectively equivalent to the larger delayed option for each delay length, which ensures that participants make an equal number of immediate and delayed choices. Immediate options are presented on the left side of the screen and are selected by pressing a button placed under the right index finger. Delayed options are presented on the right side of the screen and are selected with the right middle finger. Each trial lasts for 4 s, separated by blank screen interval of at least 8 s (depending on the participant's reaction time) (inter-trial-interval: 12 s).

In each trial of the TD task (Carlisi et al., 2016, Chantiluke et al., 2014, Christakou et al., 2011, Rubia et al., 2009) participants are presented with the choice of an amount of money (£100) available after a delay or a smaller amount of money available immediately (0–£100). Delay lengths are one week, one month and one year. For each participant, an algorithm is used to find values for the immediate option which are treated subjectively as equivalent to the larger delayed option for each delay length, thus ensuring each participant makes an equal number of immediate and delayed choices (Carlisi et al., 2016, Chantiluke et al., 2014, Christakou et al., 2011, Rubia et al., 2009). Immediate options are presented on the left side of the screen and are selected by pressing a button placed under the right index finger. Delayed options are presented on the right side of the screen and are selected with the right middle finger. Each trial lasts for 4 s, separated by blank screen interval of at least 8 s (depending on the participant's reaction time), which acts as an implicit baseline in the fMRI analysis (inter-trial-interval: 12 s). Participants complete twenty trials for each delay length (Fig. 1.). All participants completed an initial practice session of the task within a “mock scanner”. Task length is 12 min.

### 2.3 Analysis of performance data

First, indifference points were calculated for each participant at each delay length. The indifference point as defined here is the midpoint value between the lowest selected immediate reward and the next highest offered reward value, and represents the subjective value of £100 after the specified delay. The subjective value of reward on the TD task can be described using a hyperbolic decay function, and estimated using the equation V = A/(1 + *k*D), where V is the subjective value of a reward, A is size of the reward, D is the delay until reward receipt, and *k* is a constant which characterizes an individual's rate of discounting, and which is calculated by fitting a hyperbolic function to the indifference values for every delay (Christakou et al., 2011, Richards et al., 1999). Larger *k* values indicate steeper discounting (Richards et al., 1999). A three-way ANCOVA, controlling for non-significant differences in age, was performed between groups with *k* as the dependent measure to test for group differences in TD performance. To aid interpretation of potential group differences in *k,* indifference points for week, month and year delays were subjected to separate three-way ANCOVAs. Potential differences in reaction times were examined using a 2 (immediate and delayed choices) × 3 (ADHD, OCD & control groups) ANCOVA. We anticipated group differences in IQ, since ADHD is associated with low IQ (Bridgett and Walker, 2006). IQ was not covaried in the first instance as covarying for differences between groups that were not randomly selected violates ANCOVA assumptions (Miller and Chapman, 2001). However, supplementary analyses were performed covarying for IQ to test potential confounds.

### 2.4 fMRI image acquisition

The fMRI images were acquired at King's College London, Institute of Psychiatry's Centre for Neuroimaging Sciences on a 3 T General Electric Signa Horizon HDx MRI scanner (GE Healthcare, UK). Details on scanning parameters are given in the Supplement.

### 2.5 fMRI data analysis methods

Data were analysed using the non-parametric XBAM software package (Brammer et al., 1997). XBAM's non-parametric approach overcomes many of the issues associated with parametric software packages (e.g., poor control of FWE-corrected false positive cluster-wise inference rates) (Bullmore et al., 1999, Eklund et al., 2016). Details of individual and group-level analyses are described elsewhere (Christakou et al., 2009) and in the Supplement.

In short, time-series analysis of individual subject activation was performed with wavelet-based resampling (Bullmore et al., 2001a). We first convolved the task epoch of each event of interest with two Poisson model functions (4 s and 8 s delays). Using rigid-body and affine transformation, individual maps were registered into Talairach space (Talairach and Tournoux, 1988). Group maps were then produced for each experimental condition, and hypothesis testing was performed using cluster-level analysis, shown to give excellent cluster-wise type-I error control (Bullmore et al., 2001a). Time-series permutation was used to compute the distribution of the statistic of interest under the null hypothesis. The voxel-level threshold was set to *p*<0.05 to give maximum sensitivity and to avoid type-II error (Bullmore et al., 1999). Then, a cluster-mass threshold was computed from the distribution of cluster masses in the wavelet-permuted data such that the final expected number of type-I error clusters under the null hypothesis was less than one per whole brain.

For comparisons between groups, a one-way ANCOVA was conducted with group as factor and head displacement in Euclidian 3-D space and age as covariates (Bullmore et al., 2001a, Bullmore et al., 1999). Age was included as a covariate given established maturation effects on performance and neural function during TD (Christakou et al., 2011). For the between-group comparisons of the delayed-immediate contrast, less than one false activated cluster was expected at *p*<0.05 for voxel and *p*<0.027 for cluster comparisons. Analyses were repeated with IQ and *k* as additional covariates, to rule out the possibility that group differences resulted from differences in IQ or task performance.

In order to interpret the group differences in brain activation from the between-group ANCOVA, statistical measures of BOLD response (SSQ) for each participant were extracted from significant clusters, plotted, and subjected to pairwise (ADHD vs OCD, ADHD vs controls, OCD vs controls) *post-hoc t* -tests (corrected for multiple comparisons for three groups using the least significance difference method). Follow-up analyses were performed between medicated and unmedicated patients with ADHD as well as between controls, unmedicated ADHD and unmedicated OCD using these extracted BOLD responses.

## 3 Results

### 3.1 Participant characteristics

There were no significant group differences in age, but IQ was significantly lower in ADHD (Table 1).

**Table 1:** Participant characteristics.

	**Controls**	**ADHD**	**OCD**	**Sig.**	
*N*	20	26	20	–	
Medicated/unmedicated	N/A	14/12	4/16		
Age	15.3 (1.78)	14.89 (1.71)	15.75 (1.43)	*F*(2,63)=1.99, *p*=0.15	
IQ	118.9 (11.99)	102.57 (12.54)	117.7 (13.36)	*F*(2,63)=12.63, *p*<0.001	C,OCD>ADHD
SDQ Hyperactivity/Inattention	1.95 (1.58)	8.96 (1.1)	4.4 (3.03)	*F*(2,62)=71.16, *p*<0.001	ADHD>OCD>C
SDQ Emotional	0.32 (0.58)	3.62 (2.87)	4.35 (2.58)	*F*(2,62)=16.61, *p*<0.001	ADHD,OCD>C
SDQ Conduct	0.74 (1.1)	4.73 (2.47)	1.85 (1.53)	*F*(2,62)=27.54, *p*<0.001	ADHD>OCD,C
SDQ Peer	1.32 (3.07)	3.2 (2.33)	1.85 (1.90)	*F*(2,62)=4.38, *p*=0.02	C,OCD>ADHD
SDQ Prosocial	9.05 (1.78)	5.92 (2.51)	7.65 (2.58)	*F*(2,62)=11.03, *p*<0.001	ADHD>OCD,C
CY-BOCS	…	…	22.32 (5.97)		
Conner's T	…	81.12 (7.55)	…		
K mean	0.016 (0.013)	0.046 (0.042)	0.027 (0.031)	*F*(2,62)=4.61, *p*=0.01	ADHD>C,OCD
IP week	89.1 (8.72)	79.31 (18.07)	81.65 (16.37)	*F*(2,62)=2.33, *p*=0.11	
IP month	74.4 (13.69)	52.12 (28.84)	73.30 (17.97)	*F*(2,62)=7.36, *p*<0.001	C,OCD>ADHD
IP year	38.6 (23.93)	39.23 (26.42)	40.75 (25.25)	F(2,62)=0.04, p=0.96	
Immediate RT (ms)	2130.39 (596.59)	2301.14 (625.03)	2239.26 (445.1)	*F*(2,62)=0.65, *p*=0.52	
Delayed RT (ms)	2208.88 (612.48)	2310.34 (620.8)	2317.82 (418.44)	*F*(2,62)=0.23, *p*=0.8	

Abbreviations. ADHD, Attention-Deficit/Hyperactivity Disorder; CY-BOCS, Children's Yale-Brown Obsessive-Compulsive Scale; IQ, intelligence quotient; OCD, Obsessive-Compulsive Disorder; SDQ, strengths and difficulties questionnaire.

### 3.2 Performance data

ANCOVA showed a significant between-group difference in *k* (*F*(2,62)=4.49, *p*=0.02) that was driven by steeper discounting in ADHD relative to control boys (*p=*0.003) but not relative to OCD boys (*p=*0.12) (Table 1). Significant group differences were found in mean indifference points for one month delays (*F*(2,62)=7.36, *p*<0.001), which were smaller in ADHD relative to healthy controls (*p*=0.001) and patients with OCD (*p*=0.002), who did not differ from each other (*p*=0.89). This finding survived after covarying IQ (*F*(2,61)=3.44, *p*=0.04). ANCOVA comparing groups for indifference points for one week (*F*(2,62)=2.33, *p*=0.11) and one year (*F*(2,62)=0.04, *p*=0.96) were not significant. There was a trend for participants to be slower when making delayed relative to immediate choices (*F*(1,62)=3.56, *p*=0.06). However, the group by choice interaction was not significant (*F*(2,62)=0.79, *p*=0.46). Reaction times did not differ between groups for either immediate (*F*(2,62)=0.65, *p*=0.52) or delayed (*F*(2,62)=0.23, *p*=0.8) decisions. Findings remained unchanged after controlling for IQ, except that the ANCOVA for group differences in *k* remained significant only at trend level (*F*(2,61)=2.99, *p=*0.06) (Table 1).

### 3.3 fMRI data

#### 3.3.1 Movement

There were no group differences in median displacement of x, y, z rotation and translation parameters (*F*(2,63)=1.6, *p*=0.1).

#### 3.3.2 Group brain activation maps for delayed versus immediate choices

Within-group findings for delayed-immediate contrast are presented in the Supplement.

#### 3.3.3 Between-group differences

**Fig. 2:**
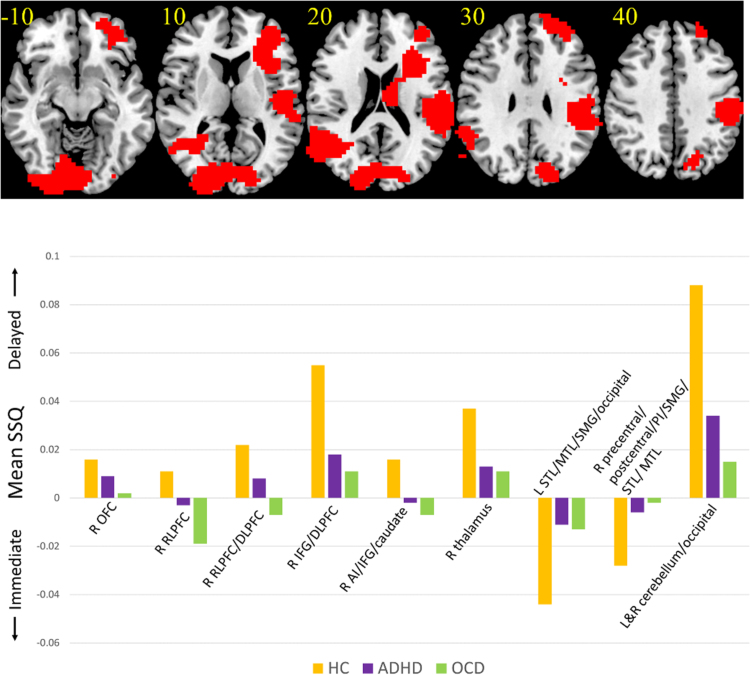
*ANCOVA results for the between-group differences in brain activation for contrast comparing delayed and immediate choices.* (A) Axial slices for the group activation maps for the three groups with a voxel threshold of *p*<0.05 and a cluster threshold of *p*<0.027. Red indicates significant between-group differences in activation for the delayed versus immediate choice contrast. Talairach z-coordinates are indicated for slice distance (in mm) from the intercommissural line. The right side of the brain corresponds to the right side of the image. (B) Bar chart showing mean SSQ for each group in each cluster. Controls = orange, ADHD = purple, OCD = green.

Whole-brain three-group ANCOVA analysis (controlling for age and motion) with follow-up *post-hoc t* -tests (corrected for multiple comparisons for three groups using least significant differences method) revealed that patients shared underactivation relative to controls in right IFG/AI/caudate (ADHD, *p*=0.02; OCD, *p*=0.006), right thalamus (ADHD, *p*=0.02; OCD, *p*=0.01) and bilateral occipital lobe/cerebellum (ADHD, *p*=0.003; OCD, *p*<0.001) during delayed relative to immediate trials, as well as left superior/middle temporal/supramarginal gyrus/fusiform gyrus (ADHD, *p*=0.001; OCD, *p*=0.003) and right postcentral/superior temporal/supramarginal gyrus/posterior insula (ADHD, *p*=0.001; OCD, *p*<0.001) to immediate relative to delayed trials. OCD patients alone showed significantly reduced activation in right OFC (*p*=0.006) and RLPFC/DLPFC (*p*=0.001) relative to controls (Table 2 and Fig. 2.). All group difference clusters remained significant after controlling for IQ and *k* using whole-brain ANCOVA. Follow-up *t*-tests on extracted statistical BOLD activation in group difference clusters performed between medicated and unmedicated ADHD patients showed a significant difference in left temporal/parietal/occipital lobe activation (*p*=0.046), which was more active during immediate choices in medicated patients. In the unmedicated subgroup analysis on extracted BOLD activation, the thalamus cluster no longer differed between ADHD and controls (*p*=0.12) and the right AI/IFG/caudate cluster differed only at a non-significant trend (*p*=0.06), presumably reflecting reduced power. All other group difference clusters remained significant. There were no significant correlations between CY-BOCS scores and brain activation in the group difference clusters in the OCD patients, or between SDQ inattention/hyperactivity scale or CPRS-R scores and brain activation in ADHD patients. There was a significant positive correlation between thalamus activation to delayed choices and *k* (*r*(26)=0.389, *p*=0.049) in ADHD patients, although this correlation did not survive correction for multiple comparisons (Benjamini and Hochberg, 1995). There were no correlations between *k* and brain activation in OCD patients or healthy controls.

**Table 2:** ANCOVA differences in brain activation between adolescents with ADHD and OCD and healthy comparison adolescents.

**Brain regions of activation**	**BA**	**TAL COORD**	**Voxels**	**Cluster*****p*****-value**
**Delay>Immediate**				
**Controls > OCD**				
R OFC	11	33,56,−18	48	0.009
R RLPFC	10/9	18,67,20	15	0.001
R RLPFC/DLPFC	46/10	29,56,26	60	0.001
**Controls >ADHD,OCD**				
R IFG/DLPFC	45/46	33,44,4	105	0.003
R AI/IFG/caudate	13/45	29,30,9	78	0.015
R thalamus		11,−11,15	27	0.01
L & R cerebellum/occipital lobe	17/18/19	−29,−96,−7	760	0.004
**Immediate>Delay**				
**Controls >ADHD,OCD**				
R precentral/postcentral/posterior insula/SMG/STL/ MTL	4/3/2/13/40/22/41/42/43	54,−15,26	332	0.01
L STL/MTL/SMG/occipital lobe	37/21/22/42/39/17/19	−51,−56, 9	232	0.02

*Abbreviations:* ADHD, Attention-Deficit/Hyperactivity Disorder; AI, anterior insula; BA, Brodmann area; DLPFC, dorsolateral prefrontal cortex; IFG, inferior frontal gyrus; MTL, middle temporal lobe; OCD, Obsessive-Compulsive Disorder; OFC, orbitofrontal cortex; RLPFC, rostrolateral prefrontal cortex; SMG, supramarginal gyrus; STL, superior temporal lobe; TAL COORD, Talairach coordinates.

## 4 Discussion

This fMRI study investigated potentially shared and disorder-specific neurofunctional abnormalities in paediatric ADHD and OCD during TD. The findings show that patient groups relative to controls, shared underactivation in key regions of self-control and temporal foresight (Noreika et al., 2013, Rubia et al., 2009), including right IFG, DLPFC, AI, dorsal striatum and bilateral cerebellum during delayed choices. Only OCD patients showed underactivation during delayed choices relative to controls in a right OFC region responsible for goal-directed reward evaluation (Christakou et al., 2011, Hare et al., 2014) and in a RLPFC region shown to mediate prospection and planning (Burgess et al., 2011, van den Heuvel et al., 2003). The findings suggest that key mechanisms associated with adaptive reward-related decision making and temporal foresight during TD are impaired in both disorders, while OFC and RLPFC regions are exclusively impaired in OCD.

During TD, IFG, DLPFC, AI, striatum and cerebellum are typically recruited more during the selection of larger delayed than immediate rewards (Christakou et al., 2011, Hare et al., 2014, Rubia et al., 2009). These regions are also activated when participants consider the negative long-term consequences of unhealthy foods (Hare et al., 2009), smoking cigarettes (Kober et al., 2010), and illegal drug use (Volkow et al., 2010), suggesting a key role in self-control and temporal foresight (Carlisi et al., 2016, Rubia et al., 2009). Findings of decreased activation in these regions extend previous findings in ADHD during TD (Ortiz et al., 2015, Rubia et al., 2009), by showing that deficits are shared with patients with OCD during this task. Interestingly, we have previously shown that IFG underactivation is disorder-specific in ADHD relative to OCD during inhibitory and attentional control (Norman et al., 2016, Norman et al., 2017, Rubia et al., 2010). Current findings are in line with suggestions that inhibitory control and TD are mediated by neuroanatomically overlapping but functionally dissociable fronto-striatal neural circuits (Fineberg et al., 2014), and suggest that IFG underactivation in ADHD relative to OCD is task-specific to the contexts of inhibitory and attentional control, but not the context of TD.

Findings of largely shared dysfunction in right IFG, DLPFC, AI, dorsal striatum and cerebellar regions during delayed choices in ADHD and OCD suggest that TD taps into a shared underlying transdiagnostic mechanism (Fineberg et al., 2014). A further implication of these findings is that shared neural deficits may potentially be normalised using the same psychological or psychopharmacological manipulation across both disorders. For instance, we recently reported that TD performance was normalised in ADHD patients relative to controls following an acute dose of the SSRI fluoxetine. This was also associated with the up-regulation of activation in right IFG, AI, and striatum, regions that we found to be underactive in ADHD and OCD in the current study (Carlisi et al., 2016). SSRIs including fluoxetine are first line treatment in OCD, and therefore it may be interesting to investigate whether shared underactivation in right hemisphere fronto-insula-striatal regions respond similarly to pharmacological manipulation across disorders.

Only OCD patients showed significantly reduced activation in right OFC during delayed choices relative to controls. The OFC is a key region for representing reward values (Hare et al., 2014), and receives signalling from both striato-limbic regions which process low-level reward properties and from DLPFC regions involved in temporal foresight and self-control, integrating both representations into a goal-directed reward valuation in order to guide long-sighted decision making (Christakou et al., 2011, Hare et al., 2014). Adults with OCD show reduced DLPFC and OFC recruitment during affective reversal (Remijnse et al., 2006b, Remijnse et al., 2009), suggesting that in OCD patients, alterations within this brain network may underlie the perseverative performance of undesired, goal-irrelevant behaviours due to a failure in flexibly updating reward associations (Remijnse et al., 2009). Findings of reduced OFC in OCD is in line with predominantly orbito-striatal accounts of the disorder (Menzies et al., 2008, Norman et al., 2016), and extends these by implicating OFC dysfunction in choice impulsivity in OCD.

In RLPFC, controls showed greater activation during delayed choices while OCD patients showed greater activation during immediate choices. RLPFC has been implicated in episodic prospection (Burgess et al., 2011), planning (van den Heuvel et al., 2003), counterfactual thinking (Boorman et al., 2011), and representing abstract, temporally extended goals (Badre and D’Esposito, 2009), i.e. in processes involved in comparing competing options and considering their long-term outcomes. Results parallel our meta-analytic finding of disorder-specific increased RLPFC grey matter in paediatric OCD relative to paediatric ADHD (Norman et al., 2016). Also, OCD patients show altered activity in this region during resting state fMRI (Le Jeune et al., 2010) and symptom provocation studies (Rotge et al., 2008). Conventional treatments including cognitive behavioural therapy (Yamanishi et al., 2009) and SSRIs (Carey et al., 2004), as well as treatment with deep-brain stimulation (Le Jeune et al., 2010) and repetitive transcranial magnetic stimulation (Nauczyciel et al., 2014) modulate RLPFC cortex activity in OCD, and targeting this region (along with adjacent OFC) with neurofeedback training is associated with a decrease in OCD symptoms (Scheinost et al., 2013, Scheinost et al., 2014). However, the nature of the relationship between RLPFC alterations and OCD is poorly understood (Gruner et al., 2016), and the findings of this study suggests that choice impulsivity may represent one mechanism linking established alterations in this region and OCD.

In line with previous research, we found evidence of steeper discounting in ADHD relative to controls (Patros et al., 2016) but unlike a previous study by Sohn and colleagues (Sohn et al., 2014), we did not find evidence of impulsive decision making in OCD patients. However, the study by Sohn and colleagues used a far larger sample size. Owing to the focus on more sensitive neural outcomes, the current study may have been underpowered to detect significant performance differences in the OCD group. It is interesting to note that significant abnormalities in brain functioning in the OCD sample in the current study are seen in the absence of significant differences in behaviour, and this finding along with a lack of any significant correlations between *k* and brain activation and the fact that differences in brain activation remained after controlling for differences in *k* suggest that there is not a one-to-one relationship between brain activation and choice behaviour during TD. As noted above, lateral prefrontal, orbitofrontal, insular and striatal regions are associated with a number of cognitive processes which may be important for TD performance, and these regions have been found to be underactive in patients with OCD during reversal learning, attentional and inhibitory control, and reappraisal tasks (de Wit et al., 2015, Norman et al., 2016, Norman et al., 2017, Remijnse et al., 2009, Rubia et al., 2010, Rubia et al., 2011). Findings may suggest a failure to appropriately recruit networks supporting these cognitive processes, which nonetheless was not enough to impact TD performance in OCD significantly. Findings of reduced activation in the absence of impaired performance are in line with previous work in paediatric OCD (Carlisi et al., 2017b, Carlisi et al., in press, Norman et al., 2017, Woolley et al., 2008).

Limitations of the study include a lower IQ in the ADHD group, in light of evidence linking IQ to TD performance (Shamosh and Gray, 2008). However, lower IQ is typical for the population (Bridgett and Walker, 2006) and findings remained significant after covarying for IQ. Second, 54% of ADHD patients were receiving psychostimulant medication which has been associated with increased fronto-striatal activation, suggesting that functional deficits in fronto-striatal systems may have been mitigated by stimulant treatment (Rubia et al., 2009). However, significant clusters remained largely unchanged between medicated and unmedicated groups, and remained significant in sub-group comparisons of unmedicated patients. While participants were determined by a consultant psychiatrist to be free of comorbidities after clinical assessment, structured interviews to assess common comorbidities including anxiety, mood, impulse and personality disorders in patients and undiagnosed conditions in controls were not performed. Relatedly, sub-clinical depression and anxiety symptoms were not assessed and were likely higher in both patient groups relative to controls (McIntosh et al., 2009; Meyer et al., 2014). Moreover, we did not assess socio-economic status (SES), and low SES has been reported to increase risk for ADHD and OCD (Heyman et al., 2001, Russell et al., 2016). This is particularly important given that depression, anxiety and low SES have been associated with steeper discounting as well as structural and functional alterations in fronto-limbic and fronto-striatal brain networks involved in reward processing and self-control (Chantiluke et al., 2012, Noble et al., 2012, Norman et al., 2015, Pulcu et al., 2014, Radua et al., 2010, Remijnse et al., 2006a, Remijnse et al., 2009, Sweitzer et al., 2013, Wise et al., 2016). Future work should aim to match groups on these clinical and demographic variables.

To summarise, the study provides the first comparison of functional abnormalities during TD between ADHD and OCD patients. Both disorders were associated with a common pattern of underactivation in fronto-striatal-insula-cerebellum regions implicated in self-control and temporal foresight during delayed choices, suggesting that choice impulsivity in both disorders may partially represent a shared transdiagnostic mechanism. In addition, we found that OFC and RLPFC were disorder-exclusively underactivated in OCD relative to controls, in line with existing orbito-striatal accounts of OCD, and providing initial evidence for its involvement in choice impulsivity in the disorder.

## Financial disclosures

K.R. has received funding from Lilly for another project and speaker's honoraria from Lilly, Shire, Novartis and Medice. C.M. has received funding from Lilly for another project and speaker's honoraria from Flynn Pharma. The other authors have no conflict of interests to declare.

## Contributions

LJN, COC, KR devised the study. LJN & COC analysed the data. LJN prepared the manuscript. LJN, COC, AC, KC, CM collected data. Included authors provided intellectual input into the final draft of the manuscript.
