# Supplementary material for: Neural dysfunction during temporal discounting in paediatric Attention-Deficit/Hyperactivity Disorder and Obsessive-Compulsive Disorder
Source: Psychiatry Res. 2017 Nov 30;269:97–105. doi: 10.1016/j.pscychresns.2017.09.008 (PMC5647646; doi:10.1016/j.pscychresns.2017.09.008)
Supplement: Supplementary file 1 — Supplementary material [file mmc1.docx]

**Supplementary Materials**

**METHODS**

***MRI image acquisition***

The fMRI images were acquired at King's College London, Institute of Psychiatry's Centre for Neuroimaging Sciences on a 3T General Electric Signa Horizon HDx MRI scanner (GE Healthcare, UK) using the body coil for radio frequency transmission and a quadrature birdcage headcoil for radio frequency transmission and reception. In each of 22 non-contiguous planes parallel to the anterior–posterior commissure, 480 T2*-weighted MR images depicting BOLD (blood oxygen level dependent) contrast covering the whole brain were acquired with echo time (TE) = 30 ms, repetition time (TR) = 1.5 s, flip angle = 60°, in-plane voxel size = 3.75 mm, slice thickness = 5.0 mm, slice skip = 0.5 mm). A whole-brain high resolution structural scan (inversion recovery gradient echo planar image) used for standard space normalisation was also acquired in the inter-commissural plane with TE = 40 ms, TR = 3 s, flip angle = 90°, number of slices: 43, slice thickness = 3.0 mm, slice skip = 0.3 mm, in-plane voxel size = 1.875 mm, providing complete brain coverage.

***fMRI Data Analysis Methods***

*Individual Analysis*

Data were first processed to minimize motion-related artefacts (Bullmore et al., 1999a). A 3-D volume consisting of the average intensity at each voxel over the entire experiment was calculated and used as a template. The 3D image volume at each time point was then realigned to this template by computing the combination of rotations (around the *x*, *y* and *z* axes) and translations (in *x*, *y* and *z*) that maximised the correlation between the image intensities of the volume in question and of the template (rigid-body registration). Following realignment, data were then smoothed using a Gaussian filter (full-width at half-maximum (FWHM) 7.2 mm) to improve the signal-to-noise ratio of the images (Bullmore et al., 1999a). Global detrending, slice timing correction, and spin-excitation history correction were performed, and the residual effects of motion were regressed out from the time series using the estimated motion parameters. Time series analysis for each subject was conducted based on a previously published wavelet-based resampling method for fMRI data (Bullmore et al., 2001; Bullmore et al., 1999b). At the individual-subject level, a standard general linear modelling approach was used to obtain estimates of the response size (beta) to each of the task conditions (delayed and immediate reward choices) against an implicit baseline. We first convolved the main experimental conditions with 2 Poisson model functions (peaking at 4 and 8s). We then calculated the weighted sum of these 2 convolutions that gave the best fit (least-squares) to the time series at each voxel. A goodness-of-fit statistic (SSQ ratio) was then computed at each voxel consisting of the ratio of the sum of squares of deviations from the mean intensity value due to the model (fitted time series) divided by that of the squares due to the residuals (original time series minus model time series). The appropriate null distribution for assessing significance of any given SSQ ratio was established using a wavelet-based data re-sampling method (Bullmore et al., 2001; Bullmore et al., 1999b) and applying the model-fitting process to the resampled data. This process was repeated 20 times at each voxel and the data combined over all voxels, resulting in 20 null parametric maps of SSQ ratios for each subject, which were combined to give the overall null distribution of SSQ ratio. This same permutation strategy was applied at each voxel to preserve spatial correlation structure in the data. Individual SSQ ratio maps were then affine transformed into standard space, by first mapping the fMRI data onto a high-resolution inversion recovery image of the same subject, and then by normalising onto a Talairach template (Talairach and Tournoux, 1988). Data were mapped to a template with a voxel size of 3.75 x 3.75 x 5.72 mm.

*Group Analysis*

A group-level activation map was produced for each group for the experimental condition (delayed versus immediate choices) by calculating the median observed SSQ ratios at each voxel in standard space across all subjects and testing them against the null distribution of median SSQ ratios computed from the identically transformed wavelet-resampled data (Brammer et al., 1997; Bullmore et al., 2001). Within-group findings are presented below, with a voxel threshold *p*<.05 and cluster threshold *p*<.05. ANCOVAs were conducted using randomization-based tests for voxel- or cluster-wise differences. The voxel-level threshold was first set to p< 0.05, as in order to maximize detection power we used the highest threshold that we have shown previously to give good type I error control at cluster level under the null hypothesis (Bullmore et al., 1999b). Next, a cluster-level threshold was computed for the resulting 3D voxel clusters in such a way as to produce less than one false positive 3D cluster per map. The necessary combination of voxel and cluster level thresholds was not assumed from theory but rather was determined by direct permutation for each dataset, giving excellent type-I and type-II error control (Bullmore et al., 1999b). Cluster mass rather than a cluster extent threshold was used to minimize discrimination against possible small, strongly responding foci of activation (Bullmore et al., 1999b). For comparisons between groups, a one-way ANCOVA analysis with group as factor and head displacement in Euclidian 3-D space and age as covariates was conducted (Bullmore et al., 2001; Bullmore et al., 1999b). In large clusters containing multiple key fronto-striatal TD regions of interest, an in-house declustering routine identified local maxima that were further apart than the upper bound of the likely Talairach mapping error (Thirion et al., 2007), and voxels were then assigned to the nearest local maximum with a statistical value that exceeded that of the voxels (Christakou et al, 2011). For between-group comparisons of the delayed-immediate contrast, less than one false activated cluster was expected at *p*<0.05 for voxel and *p*<0.027 for cluster comparisons. Analyses were repeated with IQ and *k* as additional covariates, to rule out the possibility that group differences resulted from differences in IQ or task performance.

**RESULTS**

***fMRI Data – Within-Group Activation Results***

Controls activated bilateral precunceus, postcentral gyrus, inferior parietal lobe, superior parietal lobe, supramarginal gyrus occipital lobe and cerebellum to delayed choices, and bilateral precuneus, precentral gyrus, postcentral gyrus, supramarginal gyrus, middle temporal, superior temporal lobe, posterior insula to immediate choices (Fig S1A, Supplementary Table 1).

ADHD patients activated left precentral gyrus, postcentral gyrus, parietal lobe and bilateral precuneus, occipital lobe and cerebellum to delayed choices, as well as bilateral medial prefrontal cortex, precentral gyrus and postcentral gyrus, and left caudate, supramarginal gyrus, middle temporal lobe, superior temporal lobe and occipital lobe during immediate choices (Fig S1B, Supplementary Table 1).

OCD patients activated left precentral, postcentral gyrus, bilateral inferior parietal lobe, supramarginal gyrus, occipital lobe and cerbellelum to delayed choices and bilateral medial prefrontal cortex, dorsolateral prefrontal cortex, posterior cingulate, middle temporal lobe, superior temporal lobe, occipital lobe, right caudate, precentral gyrus, postcentral gyrus, superior parietal lobe, superior temporal lobe, hippocampus, amygdala to immediate choices (Fig S1C, Supplementary Table 1).

(a)


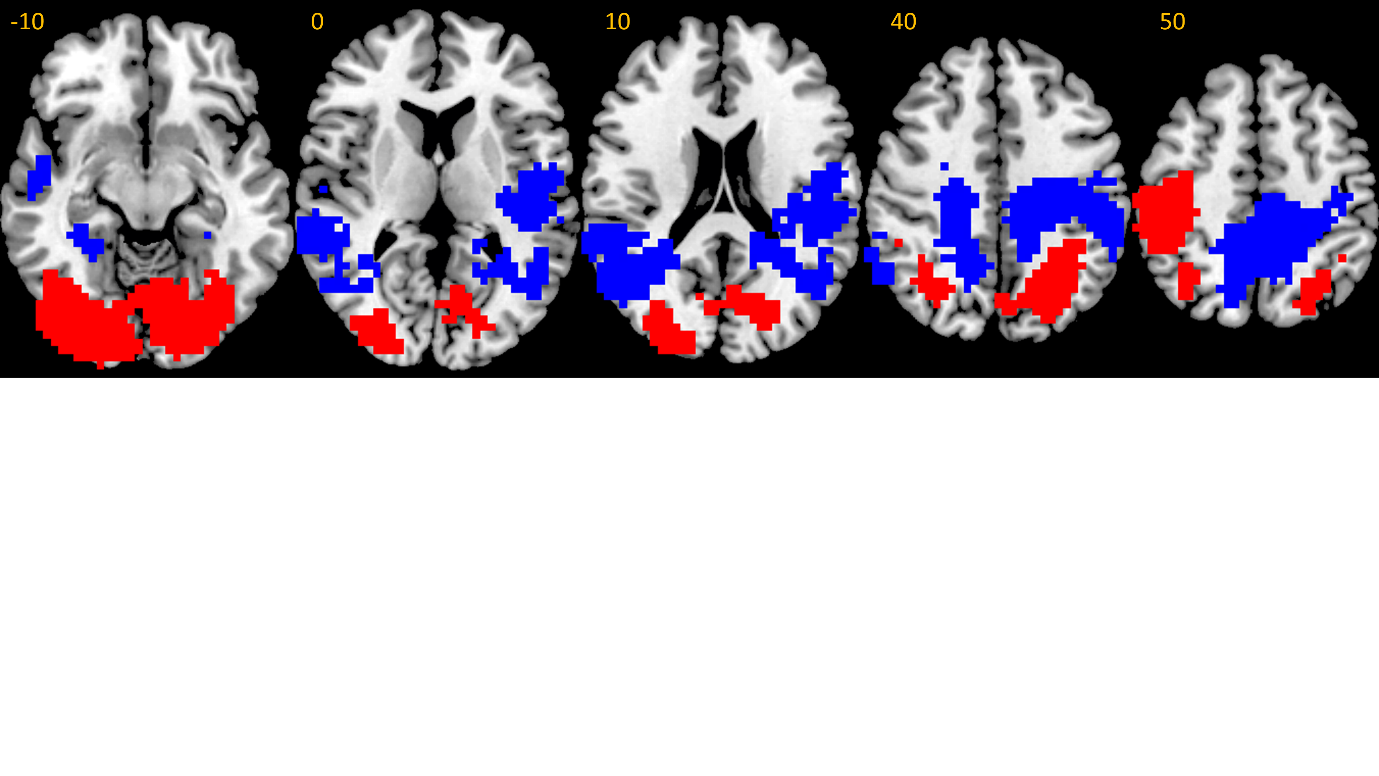


(b)


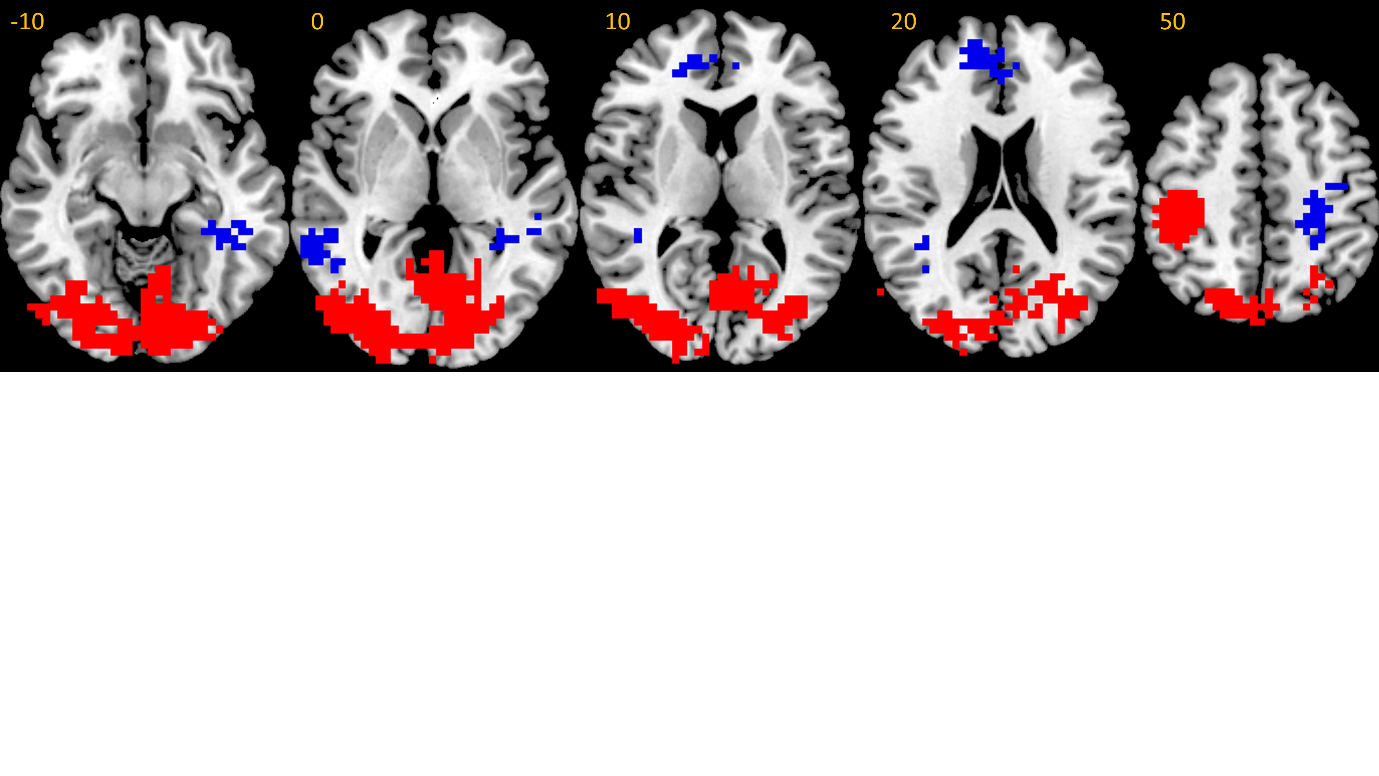


(c)


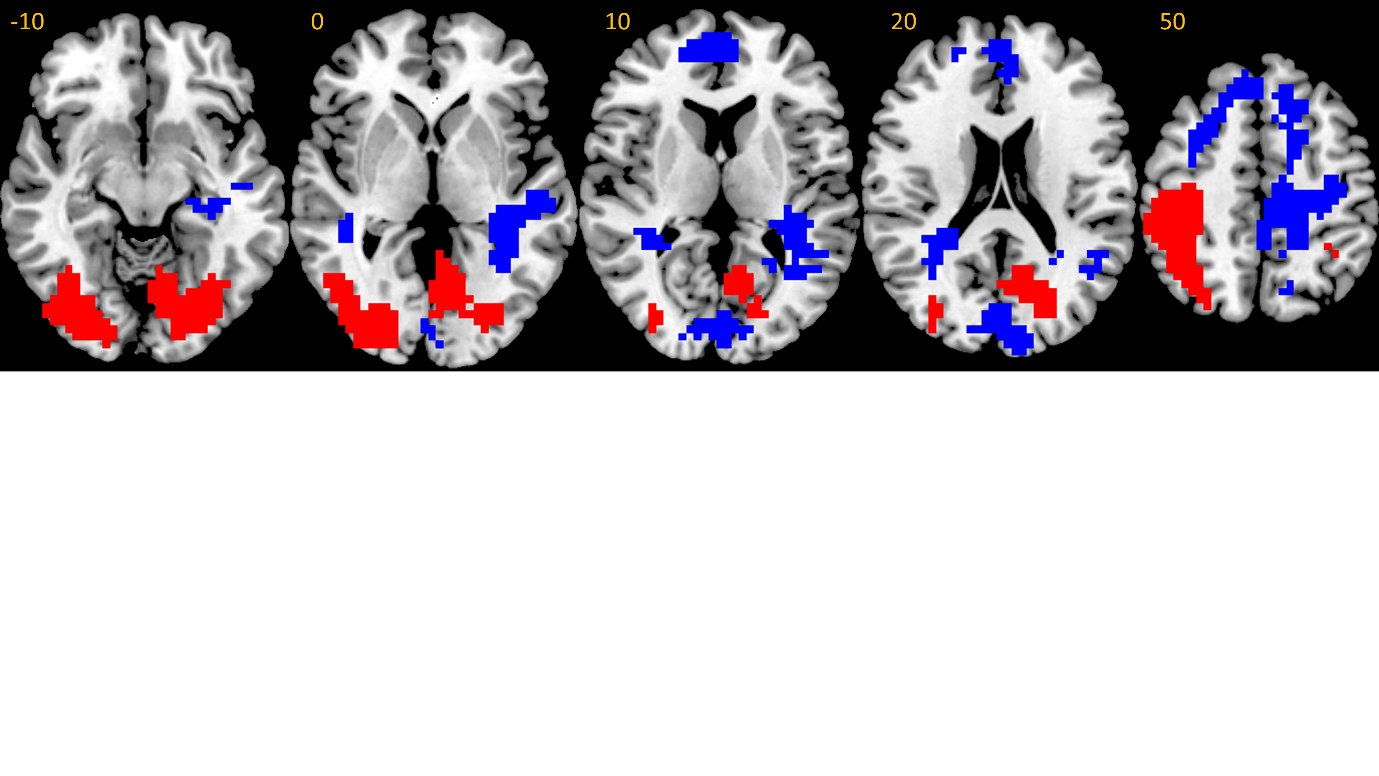


**Supplementary Figure S1.** Group activation maps. Axial slices showing within-group brain activation for the contrasts of delayed-immediate reward choices (red) and immediate-delayed reward choices (blue). (A) healthy controls, (B) ADHD patients (C) OCD patients. Talairach z-coordinates are indicated for slice distance (in mm) from the intercommissural line. The right side of the brain corresponds to the right side of the image. Data presented at voxel threshold p<.05 and cluster threshold p<.05.

**Supplementary Table 1. Group activation to delayed and immediate choices in healthy controls, adolescents with ADHD and adolescents with OCD**

| **Brain regions of activation** | **BA** | **TAL COORD** | **Voxels** | **Cluster**  **p-value** |
| --- | --- | --- | --- | --- |
| **Controls** |  |  |  |  |
| **Delay>Immediate** |  |  |  |  |
| L & R precuneus, SPL, occipital lobe, cerebellum | 7,17,18,19 | 25,-63,31 | 1353 | 0.006 |
| L & R postcentral gyrus, precentral gyrus, IPL, SPL, SMG | 3,2,4,6,40 | -40,-26,48 | 297 | 0.006 |
| **Immediate>Delay** |  |  |  |  |
| L & R precuneus, precentral gyrus, postcentral gyrus, SMG, MTL, STL, posterior insula | 7,4,5,6,40,21,22,42 | 14,-41,42 | 1815 | 0.003 |
| **ADHD** | | | | |
| **Delay>Immediate** |  |  |  |  |
| L precentral gyrus, postcentral gyrus, parietal lobe | 4,2,3,6 | -33,-26,48 | 163 | 0.002 |
| L & R occipital lobe, precuneus, cerebellum | 19,17,18,7 | -22,-78,-13 | 1333 | 0.002 |
| **Immediate>Delay** |  |  |  |  |
| R MTL | 20,21 | 47,-30,-13 | 58 | 0.036 |
| L parahippocampal gyrus, MTL | 37,21,20 | -40,-44,-2 | 43 | 0.033 |
| MPFC | 9,32,24,10 | 4,44,15 | 76 | 0.03 |
| L caudate tail, STL | 39,41 | -25,-44,15 | 32 | 0.044 |
| L precentral gyrus, postcentral gyrus | 4,3 | -51,-11,26 | 58 | 0.014 |
| L caudate |  | -14, 4, 20 | 79 | 0.02 |
| R postcentral gyrus, precentral gyrus | 3,4,6 | 25,-33,53 | 119 | 0.011 |
| **OCD** |  |  |  |  |
| **Delay>Immediate** |  |  |  |  |
| R occipital lobe, cerebellum | 19,17,18,7 | 29,-74,-13 | 572 | 0.005 |
| L occipital lobe, cerebellum, SMG, bilateral IPL, precentral gyrus, postcentral gyrus | 19,18,37,7,2,3,4,6,40 | -25,-81,-13 | 835 | 0.005 |
| **Immediate>Delay** |  |  |  |  |
| R parahippocampal gyrus, hippocampus, amygdala, MTL, STL | 19,20,21,22,37 | 36,-52,-2 | 188 | 0.016 |
| L & R occipital lobe | 18,17,19 | 4,-78,4 | 84 | 0.021 |
| L & R precentral gyrus, postcentral gyrus, PCC, precuneus, dACC, MPFC, DLPFC, R caudate | 4,6,3,5,7,32,8,24,9,10 | 29,-19,37 | 987 | 0.003 |

*Abbreviations:* ADHD, Attention-Deficit/Hyperactivity Disorder; BA, Brodmann area; dACC, dorsal anterior cingulate cortex; DLPFC, dorsolateral prefrontal cortex; IPL, inferior parietal lobe; MPFC, medial prefrontal cortex; MTL, middle temporal lobe; OCD, Obsessive-Compulsive Disorder; SMG, supramarginal gyrus; SPL, superior parietal lobe; STL, superior temporal lobe; TAL COORD, Talairach coordinates.

**Supplementary references**

Brammer, M.J., Bullmore, E.T., Simmons, A., Williams, S.C., Grasby, P.M., Howard, R.J., Woodruff, P.W., Rabe-Hesketh, S., 1997. Generic brain activation mapping in functional magnetic resonance imaging: a nonparametric approach. Magn Reson Imaging 15 (7), 763-770.

Bullmore, E., Long, C., Suckling, J., Fadili, J., Calvert, G., Zelaya, F., Carpenter, T.A., Brammer, M., 2001. Colored noise and computational inference in neurophysiological (fMRI) time series analysis: resampling methods in time and wavelet domains. Hum Brain Mapp 12 (2), 61-78.

Bullmore, E.T., Brammer, M.J., Rabe-Hesketh, S., Curtis, V.A., Morris, R.G., Williams, S.C., Sharma, T., McGuire, P.K., 1999a. Methods for diagnosis and treatment of stimulus-correlated motion in generic brain activation studies using fMRI. Hum Brain Mapp 7 (1), 38-48.

Bullmore, E.T., Suckling, J., Overmeyer, S., Rabe-Hesketh, S., Taylor, E., Brammer, M.J., 1999b. Global, voxel, and cluster tests, by theory and permutation, for a difference between two groups of structural MR images of the brain. IEEE Trans Med Imaging 18 (1), 32-42.

Christakou, A., Brammer, M., Rubia, K., 2011. Maturation of limbic corticostriatal activation and connectivity associated with developmental changes in temporal discounting. Neuroimage 54 (2), 1344-1354.

Talairach, J., Tournoux, P., 1988. Coplanar Stereotaxic Atlas of the Human Brain, a 3-Dimensional Proportional System: An Approach to Cerebral Imaging. Thieme, New York.

Thirion, B., Pinel, P., Mériaux, S., Roche, A., Dehaene, S., Poline, J.-B., 2007. Analysis of a large fMRI cohort: Statistical and methodological issues for group analyses. Neuroimage 35 (1), 105-120.
